# Association of Medicare Spending With Subspecialty Consultation for Elderly Hospitalized Adults

**DOI:** 10.1001/jamanetworkopen.2019.1634

**Published:** 2019-04-05

**Authors:** Kira L. Ryskina, Yihao Yuan, Rachel M. Werner

**Affiliations:** 1 Leonard Davis Institute of Health Economics, University of Pennsylvania, Philadelphia; 2 Division of General Internal Medicine, University of Pennsylvania, Philadelphia; 3 Center for Health Equity Research and Promotion, Corporal Michael J. Crescenz Veterans Affairs Medical Center, Philadelphia, Pennsylvania

## Abstract

**Importance:**

High use of subspecialty care is an important source of health care spending. Medical subspecialty care in particular may duplicate the scope of practice of the primary attending physicians for patients hospitalized for medical conditions. Under value-based payments, which aim to control overall spending during an episode of hospitalization (including Part B physician fees), subspecialty consultations may be a target for hospitals working to reduce costs.

**Objectives:**

To measure the use of subspecialty consultation for Medicare beneficiaries hospitalized for nonsurgical conditions; to compare payments for consultative and nonconsultative care, adjusted for case mix and demographics; and to measure variation in payments across hospital referral regions (HRRs).

**Design, Setting, and Participants:**

This retrospective cross-sectional study included a 15% random sample of Medicare fee-for-service beneficiaries enrolled in Parts A and B and identified all discharges after acute care hospital stays for nonsurgical conditions from January 1 through December 31, 2014. A total of 735 627 discharges were included. The analyses were conducted from December 1, 2017, through February 12, 2019. Total Part B payments were extrapolated to the population of Medicare fee-for-service beneficiaries.

**Main Outcomes and Measures:**

Probability of any consultation during a hospitalization was estimated using logistic regression. The number of consultations per stay and the number of consultative visits per hospital day were estimated using Poisson regression. Part B payments for consultative and nonconsultative care were estimated using generalized linear regression with gamma-log link. All models were adjusted for patient demographics and case mix. Payment models also included HRR fixed effects.

**Results:**

A total of 735 627 discharges from 4534 hospitals in 2014 were included in the analysis (41.2% men and 58.8% women; mean [SD] age, 79.6 [8.9] years; 84.7% white, 10.1% black, and 5.2% other race). After adjusting for patient case mix and demographics, a 6-fold variation between the top and bottom quintiles of hospitals (relative difference, $401 [95% CI, $368-$434]) and HRRs (relative difference, $363 [95% CI, $337-$389]) was found in payments per stay for consultative care. Part B payments for consultative care by medical subspecialists accounted for 41.3% of payments for physician visits during hospitalization and totaled $1.3 billion in 2014.

**Conclusions and Relevance:**

The substantial variation in the use of subspecialty consultative care suggests potential opportunities for cost savings.

## Key Points

**Question:**

Does inpatient consultative care by medical subspecialists represent a potential opportunity for cost savings during an episode of hospitalization for nonsurgical conditions?

**Findings:**

In this cross-sectional study of 735 627 hospital discharges among Medicare beneficiaries 65 years or older extrapolated to the population of Medicare fee-for-service beneficiaries, substantial variation in Medicare payments for medicine subspecialty consultations suggested inefficiencies. Subspecialty consultative care accounted for more than $1.3 billion dollars of Medicare spending in 2014.

**Meaning:**

Although whether patients have improved outcomes as a result of these consultations remains unclear, efforts to constrain the unnecessary use of consultative care during a hospitalization represent a potential area for savings.

## Teaser

This cross-sectional study of Medicare beneficiaries hospitalized for nonsurgical conditions measures the use of subspecialty consultations, compares payments for consultative and nonconsultative care, and measures variation in payments across hospital referral regions.

## Introduction

Value-based payment models are based on the premise that health care professionals can deliver better care at a lower cost.^1^ High use of subspecialty care is one important source of health care spending.^2,3^ In the hospital setting, subspecialty consultations are requested at the discretion of the attending physician to provide organ-system or procedural expertise.^4^ Under value-based payment models that focus on inpatient care, which aim to control overall spending during an episode of hospitalization (including Part B physician fees), subspecialty consultations may be a target for hospitals working to reduce costs.^5^ However, the costs of consultative care provided to hospitalized patients by subspecialty physicians have not been well described.

Primary care or generalist physicians have increasingly delegated more care to subspecialist physicians.^6,7,8,9,10,11^ In the ambulatory setting, the number of referrals from primary care physicians to subspecialists grew 94% between 1999 and 2009.^10^ Whether this increase reflects a necessary specialization of clinical practice in response to patient complexity or duplicative care in response to nonclinical pressures such as malpractice or productivity is not clear.^12^

Consultative care is even more common in the inpatient setting than in the ambulatory care setting. A 2010 study^13^ found that 90% of hospitalizations included at least 1 subspecialty consultation, and each hospitalization involved 2.6 subspecialties in addition to the attending or the operating physician’s specialty. That study^13^ included medical and surgical hospitalizations as well as consultations with medical and surgical specialists. However, consultations with internal medicine subspecialists for patients hospitalized for medical conditions may represent a distinct category of consultative care from surgical consultations for 2 main reasons. First, internal medicine subspecialists and generalists share similar backgrounds, including residency training and patient population, whereas general surgeons and surgical subspecialists are more narrowly focused in terms of procedures performed and patient population. Thus, medical subspecialty care may be duplicative with the scope of practice of the primary attending physician for patients hospitalized for medical conditions. Second, reimbursement to surgeons is derived largely from procedures rather than cognitive services such as consultative care. For patients who undergo surgery during their hospitalization, surgical visits are typically rolled into a global payment for the procedure and individual patient visits would not be captured in claims.

We conducted this study to evaluate inpatient consultative care by internal medicine subspecialists as a potential opportunity for cost-savings during an episode of hospitalization. Our objectives were to measure the use of subspecialty consultation for elderly adults hospitalized for nonsurgical conditions; to compare payments for consultative and nonconsultative care, adjusted for case mix and patient demographics; and to measure variation in payments for consultative care across hospitals and hospital referral regions (HRRs).

## Methods

The study was approved by the institutional review board of the University of Pennsylvania and the privacy board of the Centers for Medicare & Medicaid Services. Owing to the use of retrospective records, informed consent was waived. The reporting of this study conforms to the Strengthening the Reporting of Observational Studies in Epidemiology (STROBE) reporting guideline for cross-sectional studies.^14^

### Data and Sample

From a 15% random sample of Medicare fee-for-service beneficiaries enrolled in Parts A and B, we identified all discharges after an acute care hospital stay for nonsurgical conditions from January 1 through December 31, 2014, among beneficiaries 65 years and older. Discharges after hospitalization for surgical conditions were excluded using the diagnosis related group (DRG) code in the hospitalization claim.^15^ The study sample included 735 627 discharges from 4534 hospitals in 2014 (see eFigure in the Supplement for flowchart of sample selection). Professional claims were linked to hospitalization claims using dates of service and place of service codes. Hospital characteristics were obtained from the Medicare Provider of Service file.

### Identification of Subspecialty Consultations

We used 2 specifications to measure consultative care. The first specification included only those stays where the attending physician was a generalist (internal medicine, general/family practice, or geriatrics) and defined any visits by subspecialists during the hospital stay as consultative care. We first identified those hospitalizations where the attending physician was a generalist based on the specialty code for the evaluation and management claims corresponding to the hospital visit, including claims for patients admitted under observational care.^16,17^ For those hospitalizations, we identified all evaluation and management services performed by subspecialists (in the fields of allergy/immunology, cardiology, gastroenterology, pulmonology, nephrology, infectious disease, endocrinology, rheumatology, critical care, hematology, hematology/oncology, preventive medicine, and medical oncology) during the hospital stay. Hospital stays without any generalist visits were excluded from this analysis (9.4% of discharges).

Although most attending physicians for patients hospitalized for medical conditions are generalists, patients can be hospitalized on a subspecialist service where the attending physician is a subspecialist who could consult other subspecialists. To account for this possibility, we conducted a second set of analyses that included hospitalizations where the attending physician was a specialist. To do this, we identified hospitalizations without any generalist visits and counted the number of visits by each medicine subspecialty. Next, we assigned the hospitalization to the subspecialty that submitted a plurality of claims for patient visits as the attending subspecialty. Physicians who belonged to other subspecialties were considered consultants, and their services and payments were summed to measure use of consultative care and costs for these hospitalizations.

### Variables

To measure the use of subspecialty consultation, we counted the number of hospital visits by physicians in each subspecialty. This variable was defined as any line items for hospital-based evaluation and management services submitted during the hospital stay by physicians in each subspecialty. We measured use of consultative care in 2 ways. First, we measured the number of consultations per hospital stay as the number of distinct subspecialties with at least 1 consultation day during a hospital stay. Second, because generalist and subspecialty physicians have broad discretion about how frequently they see patients during the hospital stay, we calculated the number of consultative visits per hospital day as the total number of consultation days for all specialties divided by the length of hospital stay.

We also measured Medicare payments associated with consultative care. To do so, Part B payments associated with the consultative visits were aggregated at the hospital-stay level. Nonconsultative visits and Part B payments for the primary attending services were similarly measured at the hospital-day and hospital-stay levels. In the first set of analyses, generalists provided nonconsultative services by definition. In the second set of analyses, which included stays where the primary attending physician was a subspecialist, nonconsultative services were provided by a generalist or by the subspecialist with the plurality of claims.

### Statistical Analysis

The analyses were conducted from December 1, 2017, through February 12, 2019. We compared discharge characteristics using χ^2^ tests for categorical variables and unpaired *t* tests for continuous variables. *P* < .05 was considered statistically significant; all hypothesis tests were 2-sided. The total payments for consultative and nonconsultative care from the 15% random sample were extrapolated to the entire Medicare fee-for-service population of Medicare beneficiaries 65 years and older.

Probability of any consultation during a hospitalization was estimated using logistic regression. The number of consultations per stay and the number of consultative visits per hospital day were estimated using Poisson regression. Payments were estimated using generalized linear regression with gamma-log link. All models were adjusted for patient demographics (age at admission, sex, and race), Elixhauser comorbidities, any time spent in the intensive care unit,^18^ and whether the patient died in the hospital. We also included dummies for the primary DRG for the hospitalization (DRGs of different severity levels were coded separately). Payment models also included HRR fixed effects to account for geographic differences in reimbursement rates. We omitted observations with 1 or more missing variables from analysis (33 discharges were missing hospital characteristics [<0.001% of observations]). The Huber-White sandwich estimator was used in all regressions to account for clustering of observations within hospitals.^19^

To describe variation in consultative care by hospital and market characteristics, we used logistic regression to estimate mean adjusted probability of a subspecialty consultation as a function of hospital and market characteristics. In addition to the patient demographic and clinical characteristics, independent variables included in this model were hospital size (<100, 100-399, or ≥400 beds), ownership (private for-profit, private not-for-profit, religious organization, federal, state, or local government, or other [eg, physician]), rural vs urban location, and teaching status (any residents), as well as 2 market characteristics. Markets were characterized at the HRR level by measuring the health maintenance organization (HMO) penetration rate and hospital market competition within each HRR. The HMO penetration rate was estimated by calculating the proportion of all Medicare beneficiaries who were enrolled in Medicare Advantage using the Medicare Beneficiary Summary file. The hospital market competitiveness within each HRR was measured by calculating the Herfindahl-Hirschman index, which is the sum of the squares of each hospital’s share of patients in the HRR. The HRRs were categorized into quartiles for HMO penetration rate and Herfindahl-Hirschman index for ease of interpretation. Statistical analyses were performed using Stata software (version 15; StataCorp).

## Results

A total of 735 627 discharges from 4534 hospitals in 2014 were analyzed (41.2% men and 58.8% women; mean [SD] age, 79.6 [8.9] years; 84.7% white, 10.1% black, and 5.2% other race) (Table 1). Compared with stays without consultative care, a larger proportion of hospital stays with consultative care took place in hospitals located in the Northeast (22.1% vs 16.2%; *P* < .001) and those that were large (38.9% vs 28.3%; *P* < .001), urban (90.0% vs 71.5%; *P* < .001), and teaching (42.9% vs 34.0%; *P* < .001). The median hospital length of stay was 5 days (interquartile range [IQR], 4-8 days) for stays with consultative care and 4 days (IQR, 3-5 days) for stays without consultative care.

**Table 1.** Patient and Hospital Characteristics of Discharges After Hospitalization for Nonsurgical Conditions by Medicare Fee-for-Service Beneficiaries in 2014^a^

Characteristic	Type of Hospital Stay		
	All	With Consultative Care^b^	Without Consultative Care^a^
Patient sex, %			
Male	41.2	43.5	38.5
Female	58.8	56.5	61.6
Patient age, mean (SD), y	79.6 (8.9)	78.9 (8.8)	80.5 (8.8)
Patient race, %			
White	84.7	83.0	86.7
Black	10.1	11.4	8.6
Other	5.2	5.6	4.7
Hospital region, %			
Northeast	19.4	22.1	16.2
South	41.6	42.1	41.1
Midwest	24.7	23.6	26.0
West	14.3	12.3	16.8
Hospital size, %			
Small	13.6	5.7	23.0
Medium	52.4	55.4	48.7
Large	34.1	38.9	28.3
Hospital type. %			
Not-for-profit private	48.0	48.6	47.2
For-profit private	15.0	16.4	13.5
Religious organization	11.6	12.5	10.5
Government	12.0	11.9	12.0
Other	13.5	10.6	16.8
Hospital location, %			
Urban	81.5	90.0	71.5
Rural	18.5	10.0	28.6
Hospital teaching status, %			
No	61.2	57.1	66.0
Yes	38.8	42.9	34.0
Hospital length of stay, median (IQR), d	5 (3-7)	5 (4-8)	4 (3-5)
No. of specialties consulted per stay, %			
None	47.3	NA	100.0
1	31.7	59.4	NA
2	13.5	25.8	NA
≥3	7.6	14.8	NA

Abbreviations: IQR, interquartile range; NA, not applicable.

^a^ Percentages have been rounded and may not total 100.

^b^ All comparisons between stays with and without consultative care are significant at *P* < .001.

In 2014, 52.8% of all discharges after hospitalization for nonsurgical conditions and 54.5% of discharges where the attending was a generalist included at least 1 consultation with a medicine subspecialist. Of all hospitalizations for nonsurgical conditions, 31.7% included a consultation with 1 subspecialty; 13.5%, 2 subspecialties; and 7.6%, 3 or more subspecialties (Table 1).

Table 2 shows the use of specialties consulted per stay, consultative visits per hospital day, and Medicare Part B payments. Substantial variation across stays occurred in the use of consultative care. For stays with a generalist attending, a median of 0.78 different specialties was consulted per hospital stay (IQR, 0.51-1.13), with a median of 0.40 consultative care visits per day (IQR, 0.26-0.57). On the other hand, little variation across stays occurred in the number of nonconsultative care visits per hospital day (median, 0.87; IQR, 0.85-0.88). These finding were stable to including stays where the attending physician was a specialist (Table 2).

**Table 2.** Number of Specialists Consulted per Stay, Number of Consultative Visits per Hospital Day, and Medicare Part B Payments, Adjusted for Case Mix and Demographics, 2014

Utilization Measures	Type of Hospital Stay, Median (IQR)^a^			
	With Generalist Attending		With Generalist or Subspecialty Medicine Attending	
	Consultative Care	Nonconsultative Care	Consultative Care	Nonconsultative Care
No. of consultations per stay^b^	0.78 (0.51-1.13)	NA	0.75 (0.50-1.08)	NA
No. of visits per hospital day^c^	0.40 (0.26-0.57)	0.87 (0.85-0.88)	0.38 (0.25-0.54)	0.86 (0.84-0.87)
Part B spending, $				
Per stay	156 (75-324)	357 (294-441)	151 (74-306)	351 (289-434)
Per hospital day	28 (15-53)	73 (55-88)	27 (15-50)	71 (66-76)

Abbreviations: IQR, interquartile range; NA, not applicable.

^a^ Based on a 15% random sample of 666 388 stays in 4528 hospitals where the attending physician was a generalist and 735 627 stays in 4534 hospitals where the attending physician was a generalist or a specialist.

^b^ Indicates the number of distinct subspecialties with at least 1 consultation visit during a hospital stay.

^c^ Indicates the total number of consultation days for all specialties divided by the length of stay.

Table 3 shows the adjusted probability and odds of consultation by hospital and market characteristics. After adjusting for case mix, stays at hospitals in the Northeast had higher odds of a subspecialty consultation compared with hospitals located in the Midwest (odds ratio [OR], 0.74; 95% CI, 0.67-0.83), South (OR, 0.81; 95% CI, 0.73-0.90), or West (OR, 0.41; 95% CI, 0.36-0.47). Stays at small hospitals had lower odds of a subspecialty consultation (OR, 0.35; 95% CI, 0.31-0.39) compared with large hospitals. Stays at hospitals with private not-for-profit ownership (OR, 0.74; 95% CI, 0.68-0.81) or government hospitals (OR, 0.62; 95% CI, 0.55-0.69) had lower odds of consultation compared with hospitals owned by private for-profit organizations. Stays at hospitals in urban locations had higher odds of consultative care (OR, 2.53; 95% CI, 2.31-2.78) compared with hospitals in rural locations. The odds of consultation were not significantly different between teaching and nonteaching hospitals (OR, 0.97; 95% CI, 0.90-1.05). These findings were generally stable to including stays where the attending was a subspecialist (Table 3).

**Table 3.** Probability of Consultation by Hospital and Market Characteristics, Adjusted for Case Mix and Demographics, 2014

Characteristic	Hospital Stay With Generalist Attending Physician		Hospital Stay With Generalist or Subspecialty Medicine Attending Physician	
	Probability of Consultation, Mean % (95% CI)^a^	OR (95% CI)^a^	Probability of Consultation, Mean % (95% CI)^a^	OR (95% CI)^a^
Region				
Northeast	59.8 (58.2-61.4)	1 [Reference]	58.0 (56.4-59.7)	1 [Reference]
Midwest	54.3 (53.1-55.5)	0.74 (0.67-0.83)	52.4 (51.2-53.6)	0.75 (0.67-0.83)
South	55.9 (54.9-56.9)	0.81 (0.73-0.90)	54.1 (53.1-55.2)	0.82 (0.74-0.91)
West	43.1 (41.4-44.7)	0.41 (0.36-0.47)	41.7 (40.1-43.4)	0.44 (0.39-0.50)
Hospital size, No. of beds				
≥400	57.1 (55.8-58.4)	1 [Reference]	54.2 (52.9-55.5)	1 [Reference]
100-399	56.7 (55.9-57.6)	0.98 (0.91-1.07)	55.4 (54.5-56.2)	1.06 (0.98-1.15)
<100	36.5 (34.6-38.3)	0.35 (0.31-0.39)	35.6 (33.7-37.4)	0.40 (0.35-0.45)
Type of hospital				
Private for-profit	59.0 (57.6-60.4)	1 [Reference]	57.3 (55.8-58.8)	1 [Reference]
Private not-for-profit	53.4 (52.5-54.4)	0.74 (0.68-0.81)	51.8 (50.8-52.8)	0.76 (0.69-0.83)
Religious organization	56.7 (54.9-58.5)	0.88 (0.78-1.00)	55.2 (53.4-57.0)	0.90 (0.80-1.01)
Government	50.1 (48.5-51.7)	0.62 (0.55-0.69)	48.1 (46.4-49.7)	0.63 (0.56-0.70)
Other	55.0 (53.2-56.8)	0.80 (0.71-0.91)	53.3 (51.4-55.1)	0.81 (0.72-0.92)
Location				
Rural	39.4 (37.8-41.0)	1 [Reference]	38.2 (36.6-39.8)	1 [Reference]
Urban	57.5 (56.8-58.2)	2.53 (2.31-2.78)	55.5 (54.8-56.3)	2.35 (2.15-2.57)
Teaching status				
No	54.7 (53.8-55.5)	1 [Reference]	53.2 (52.4-54.0)	1 [Reference]
Yes	54.1 (53.0-55.2)	0.97 (0.90-1.05)	52.1 (51.0-53.2)	0.94 (0.41-1.00)
HMO penetration rate, quartile^b^				
Lowest	52.4 (51.1-53.8)	1 [Reference]	50.6 (49.2-52.0)	1 [Reference]
Second	52.2 (51.0-53.4)	0.99 (0.90-1.09)	50.5 (49.3-51.8)	1.00 (0.91-1.09)
Third	55.8 (54.5-57.0)	1.19 (1.08-1.32)	54.1 (52.8-55.3)	1.19 (1.08-1.31)
Highest	57.3 (56.0-58.6)	1.29 (1.17-1.43)	55.7 (54.3-57.0)	1.29 (1.17-1.43)
Market concentration, quartile^c^				
Lowest	56.5 (55.4-57.6)	1 [Reference]	54.3 (53.2-55.4)	1 [Reference]
Second	55.8 (54.4-57.2)	0.96 (0.87-1.06)	54.2 (52.7-55.6)	0.99 (0.91-1.09)
Third	53.2 (51.9-54.5)	0.84 (0.77-0.92)	51.8 (50.4-53.1)	0.88 (0.81-0.96)
Highest	52.3 (51.0-53.5)	0.80 (0.73-0.87)	50.7 (49.5-52.0)	0.84 (0.77-0.91)

Abbreviations: HMO, health maintenance organization; OR, odds ratio.

^a^ Adjusted for patient case mix and demographics.

^b^ Measured as the proportion of Medicare beneficiaries in each hospital referral region who were enrolled in Medicare Advantage in 2014. The mean rate in each quartile was 13.7% in the lowest quartile, 23.3% in the second quartile, 30.9% in the third quartile, and 43.1% in the highest quartile.

^c^ Measured using Herfindahl-Hirschman Index (calculated as the sum of each hospital’s share of the patient population in the hospital referral region). The mean Herfindahl-Hirschman Index in each quartile was 0.04 in the lowest quartile of hospital market concentration, 0.08 in the second quartile, 0.14 in the third quartile, and 0.32 in the highest quartile of market concentration.

Hospitalizations in HRRs in the highest 2 quartiles of HMO penetration rate had higher odds of consultative care (OR for the third quartile, 1.19 [95% CI, 1.08-1.32]; OR for the highest quartile, 1.29 [95% CI, 1.17-1.43]) compared with the lowest quartile of HMO penetration rate (Table 3). The highest 2 quartiles of hospital market concentration were associated with lower odds of consultative care (OR for the third quartile, 0.84 [95% CI, 0.77-0.92]; OR for the highest quartile, 0.80 [95% CI, 0.73-0.87]) compared with the lowest quartile of market concentration (Table 3). These findings were also stable to including stays where a subspecialist was the primary attending physician (Table 3).

**Figure 1.**
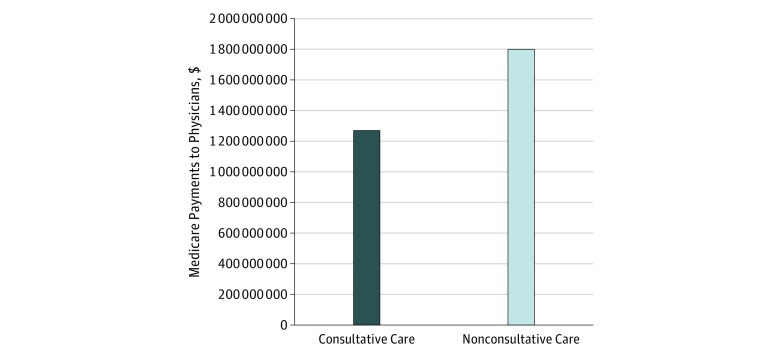
Difference in Total Part B Spending for Inpatient Consultative vs Nonconsultative Care Participants include Medicare fee-for-service beneficiaries hospitalized for nonsurgical conditions, adjusted for case mix and demographics, January 1 through December 31, 2014. Total payments are extrapolated to the Medicare fee-for-service population. In 2014, total Part B payments for patients who were hospitalized for nonsurgical conditions were $1.3 billion for consultative care and $1.8 billion for nonconsultative care.

Medicare Part B payments were more variable for consultative care than for nonconsultative care. The median adjusted Part B payment for consultative care was $156 (IQR, $75-$324) per stay and $28 per hospital day (IQR, $15-$53) (Table 2). The median adjusted Part B payments for nonconsultative care were $357 (IQR, $294-$441) per stay and $73 per hospital day (IQR, $55-$88) (Table 2). Adjusted for case mix, consultative care accounted for 41.3% of total Medicare Part B payments for internal medicine physician visits during acute care hospitalizations. In 2014, total Part B payments for consultative care provided to Medicare fee-for-service beneficiaries during hospitalization for nonsurgical conditions were $1.3 billion, whereas total Part B payments for nonconsultative care provided during these hospitalizations were $1.8 billion (Figure 1).

**Figure 2.**
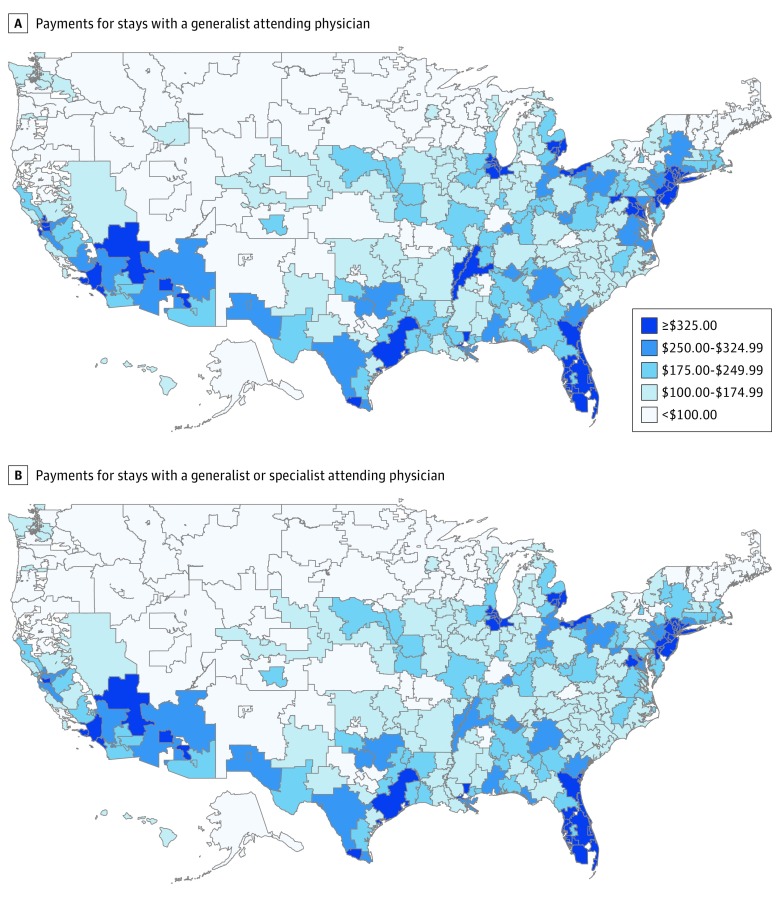
Mean Medicare Part B Payments for Consultative Care per Stay by Hospital Referral Region Data include Medicare fee-for-service beneficiaries hospitalized for nonsurgical conditions, January 1 through December 31, 2014. Mean payments by hospital referral region are adjusted for case mix and demographics.

Substantial variation occurred across hospitals and HRRs in payments for consultative care. Across HRRs, the risk-adjusted Part B payments for consultative care varied approximately 6-fold between the top and bottom quintiles of payments (Figure 2). The relative difference in payments per stay between the HRRs in the highest vs lowest quintiles of Part B payments for consultative care was $363 (95% CI, $337-$389). Payments for consultative care varied similarly (6-fold) between the top and bottom quintiles of hospitals. The relative difference in payments per stay between the hospitals in the highest vs lowest quintiles of Part B payments for consultative care was $401 (95% CI, $368-$434).

## Discussion

We found substantial variation in Medicare payments for medicine subspecialty consultations. The regional variation in the use of consultative care suggests that the practice of consulting subspecialists during a hospitalization could be optimized. Overall, we found that subspecialty consultative care accounted for $1.3 billion dollars of Medicare spending in 2014.

Our estimate of Medicare spending is large but is likely an underestimate, because we include only admissions for medical DRGs. In addition, likely substantial downstream costs are associated with these inpatient subspecialty consultations, including additional diagnostic testing conducted during or after the hospitalization and subsequent outpatient follow-up visits. A recent study found that inpatient consultation after total joint arthroplasty was associated with higher total use of services compared with cases with similar comorbidities, complication rates, and length of stay that did not involve a consultation.^20^ Furthermore, consultative care may prolong length of stay if patients are required to remain hospitalized while awaiting results of diagnostic tests requested by the consultant and to make arrangements for the additional outpatient follow-up appointments. Aggregating prospective payments that do not account for length of stay does not fully capture costs to hospitals from prolonged hospitalization.

Consistent with prior studies,^11^ we observed considerable variation in consultation rates by regional and hospital characteristics. However, identifying potentially unnecessary consultations can be challenging due to a lack of universal guidelines for consultation.^21,22^ Prior literature also suggests that some consultations save money in the long run. For example, inpatient palliative care consultation programs have been shown to save hospitals money and to provide improved care to patients with serious illness.^23^ Furthermore, some variation in consultation rates may stem from underuse as well as overuse. For instance, regional variation in physician supply results in rural hospitals having trouble attracting physicians in some subspecialties. Consistent with other analyses of regional variation in Medicare spending, regional differences in specialist involvement in care were attenuated after adjusting for patient case-mix.^24,25^

At the HRR level, higher rates of HMO penetration and more competitive markets for hospital care were associated with the use of consultative care. These findings are somewhat counterintuitive. Medicare Advantage plans receive lump sum payments from Medicare to encourage more efficient use of health care resources, such as consultative care. Similarly, one might expect lower rates of use of consultative care in more competitive markets, as hospitals attempt to reduce costs to increase market share. One possible explanation is that hospitals are primarily reimbursed on a fee-for-service basis measured by DRG. Thus, the incentive to reduce length of stay outweighs other cost considerations. If hospitals (or the attending physicians who act as hospital agents in the use of consultations and length of stay) view consultative care as a potential way to reduce length of stay, then we would expect higher consultation rates in regions with higher HMO penetration and market competition. In general, physician services are much less costly to hospitals than the opportunity cost of another inpatient stay. Other possible explanations include variation in physician supply across HRRs, which may confound the association between market characteristics and use of consultative care. Moreover, an inverse relationship may occur between high consultative care use (or overuse) and higher rates of HMO penetration and market competition.

Nevertheless, optimizing use of consultative care may be an attractive strategy for hospitals seeking to constrain costs during an episode of hospitalization. This situation may be particularly true for physician-led accountable care organizations that may be better able to achieve efficiencies by identifying and eliminating unnecessary consultations and optimizing use of subspecialists during an episode of care. Although ambulatory subspecialty care can originate from patient self-referral and is associated in part with patient insurance coverage and income,^26^ inpatient consultation is typically under the control of the attending physician. More direct involvement of those physicians with value-based payments (such as via physician-led accountable care organizations) may more effectively counteract the considerations that lead to overuse of consultative care (eg, malpractice concerns or clinical inertia).

Whether hospitals transitioning to value-based payment can reduce spending on consultative care without detrimentally affecting quality of care is unknown. Well-designed value-based payments align financial incentives across a diverse group of health care providers, including physicians, hospitals, and post–acute care providers. Such alignment should encourage health care organizations to shift resources from more expensive settings (eg, hospitals) to less expensive settings (eg, physician offices) and help limit overused services. However, value-based payments based on episodes of care also lead providers to avoid high-risk patients or focus care away from high-cost services. Recent evaluations found little empirical evidence of adverse effects of value-based payments on the quality of hospital care,^27,28^ Nevertheless, quality and outcomes of consultative care may be difficult to evaluate. For example, the association between inpatient consultative care use and existing measures of hospital quality, such as 30-day mortality and readmissions, is not well understood. On the one hand, more liberal use of inpatient consultative care may improve these outcomes by expediting patient access to higher-intensity care, quicker diagnostic workup, and more advanced techniques. On other hand, involvement of multiple subspecialty consultants may increase risks associated with care fragmentation and iatrogenic risks owing to prolonged hospitalization. Future studies should evaluate outcomes associated with different levels of use of inpatient consultative care.

### Limitations

This study has several limitations. First, we measured direct payments to physicians and did not consider other costs to the hospital and payers associated with consultative care, such as downstream costs of additional diagnostic testing, secondary consultations, and prolonged length of stay. As a result, our cost estimates of consultative care likely underestimate the total costs. Second, we did not measure quality of the care or associated outcomes and were therefore unable to evaluate the relative value of consultative care during the hospitalization. Third, variation in spending on generalist care was limited in part by Medicare regulations that require attending physicians to submit no more than 1 visit claim per hospital day, whereas different specialists can bill separately on the same day.

## Conclusions

Direct costs to Medicare attributable to inpatient subspecialty consultations for medical conditions accounted for $1.3 billion in 2014. Whether patients have improved outcomes as a result of these consultations remains unclear, but the substantial variation in the use of subspecialty consultative care suggests potential opportunities for cost savings. Efforts to identify and constrain the unnecessary use of consultative care during a hospitalization may represent a potential area for savings under value-based reimbursement.

## Notes

Supplement. **eFigure.** Study Sample Selection Flowchart
