# Supplementary material for: Association of Medicare Spending With Subspecialty Consultation for Elderly Hospitalized Adults
Source: JAMA Netw Open. 2019 Apr 5;2(4):e191634. doi: 10.1001/jamanetworkopen.2019.1634 (PMC6450334; doi:10.1001/jamanetworkopen.2019.1634)
Supplement: Supplement. — eFigure. Study Sample Selection Flowchart [file jamanetwopen-2-e191634-s001.pdf]

Supplementary Online Content

Ryskina KL, Yuan Y, Werner RM. Association of Medicare spending with subspecialty consultation for elderly hospitalized adults. *JAMA Netw Open*. 2019;2(4):e191634. doi:10.1001/jamanetworkopen.2019.1634

eFigure. Study Sample Selection Flowchart

This supplementary material has been provided by the authors to give readers additional information about their work.

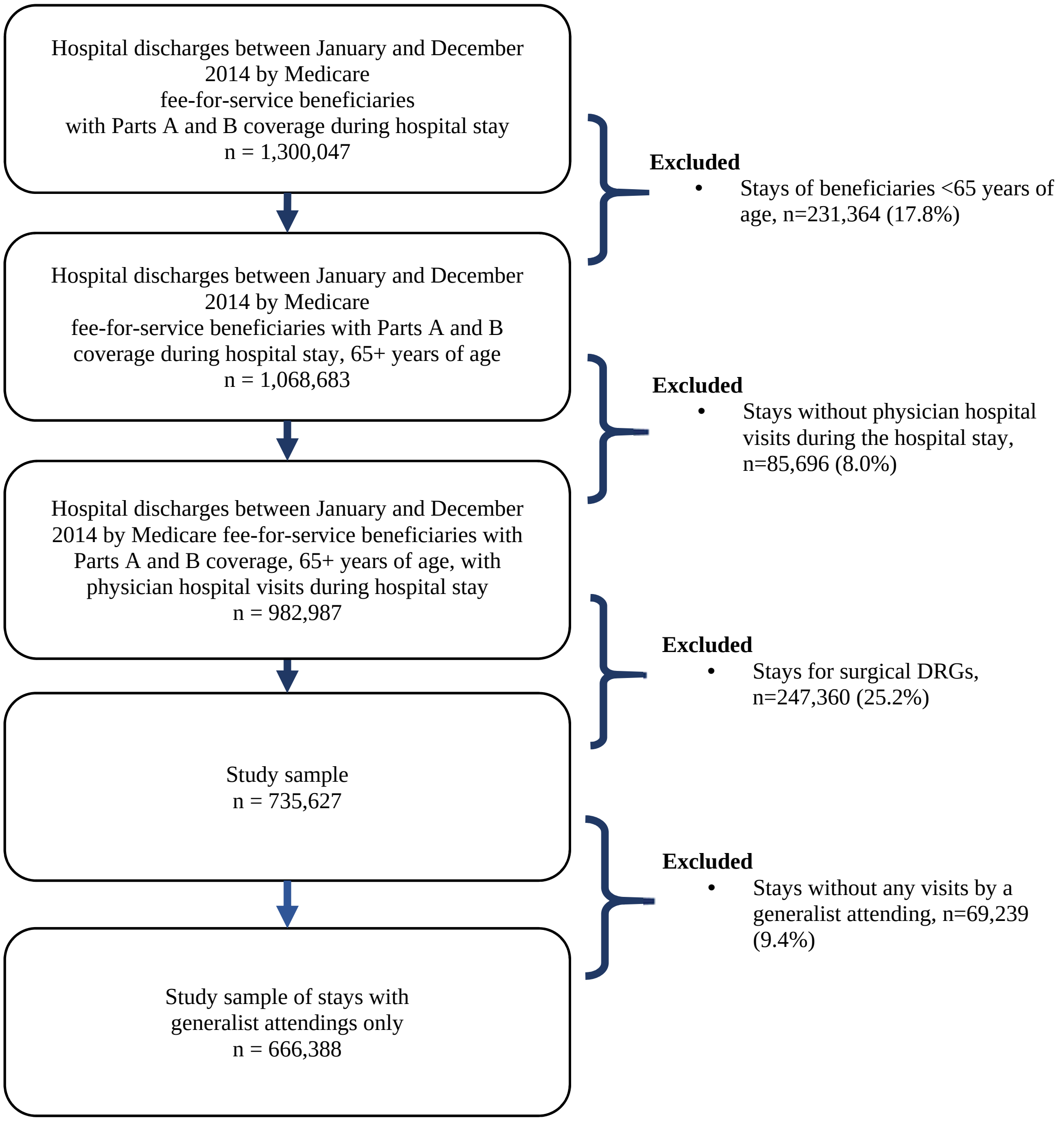

eFigure. Study Sample Selection Flowchart

© 2019 Ryskina KL et al. *JAMA Network Open.*
